# Effect of Beta 2-Adrenergic Receptor Gly16Arg Polymorphism on Taste Preferences in Healthy Young Japanese Adults

**DOI:** 10.3390/nu14071430

**Published:** 2022-03-29

**Authors:** Kohei Narita, Tada-aki Kudo, Guang Hong, Kanako Tominami, Satoshi Izumi, Yohei Hayashi, Junichi Nakai

**Affiliations:** 1 Division of Oral Physiology, Tohoku University Graduate School of Dentistry, Sendai 980-8575, Japan; kohei.narita.a2@tohoku.ac.jp (K.N.); tominami@tohoku.ac.jp (K.T.); satoshi.izumi.b8@tohoku.ac.jp (S.I.); junichi.nakai.a5@tohoku.ac.jp (J.N.); 2 Graduate Medical Education Center, Tohoku University Hospital, Sendai 980-8574, Japan; 3 Division of Globalization Initiative, Liaison Center for Innovative Dentistry, Tohoku University Graduate School of Dentistry, Sendai 980-8575, Japan; hong.guang.d6@tohoku.ac.jp; 4 Cell Resource Center for Biomedical Research, Institute of Development, Aging and Cancer, Tohoku University, Sendai 980-8575, Japan; yohei.hayashi.e2@tohoku.ac.jp; 5 Graduate School of Life Sciences, Tohoku University, Sendai 980-8577, Japan

**Keywords:** beta 2-adrenergic receptor (*ADRB2*), fatty foods, obesity, preference of food, single nucleotide polymorphism (SNP)

## Abstract

The Gly16Arg polymorphism results in a G to C nucleotide mutation in the human beta 2-adrenergic receptor (*ADRB2*) gene and has a relationship with obesity; however, this substitution’s effects on food preferences are unclear. Therefore, we determined this relationship among healthy young adults (mean age, 23.4; *n* = 52). To evaluate food preferences, four categories of food (sweet, salty, sour, and bitter) along with high-fat foods were evaluated using a self-reporting questionnaire. Male (*n* = 26) and female subjects (*n* = 26) were genotyped for the polymorphism and further divided into three groups (two homozygous groups, GG, CC; and a heterozygous group, GC). Preference for sour foods in the GG group was higher compared with that in the CC group in females (*p* < 0.05). When sweet foods were classified into low- and high-fat subgroups, preference for high-fat sweet foods in the GG group was higher than that for low-fat sweet foods in all subjects (*p* < 0.05). The degree of preference for high-fat foods in the GG group was higher than other groups for males (*p* < 0.05). These results suggest that *ADRB2* polymorphism is associated with food preference. Understanding the relationship of *ADRB2* substitution to food preference will be valuable for designing individualized anti-obesity strategies.

## 1. Introduction

Obesity significantly decreases one’s overall life expectancy and health-related quality of life [1,2]. It is an important public health concern and a global epidemic [3,4,5]. In fact, obese individuals are at increased risk of cancer, hyperlipidemia, coronary heart disease, stroke, and type II diabetes [6,7,8,9,10]. Excess food intake is a major contributor to obesity [5,11], and evidence has shown that stress induces the consumption of palatable food, which further contributes to obesity [12].

Because the consumption of palatable foods is an instinctive behavior that is required for maintaining homeostasis, and may satisfy and relax individuals mentally [13], it is not easy to simply enact dietary restrictions for a long period of time in individuals to improve health. Therefore, it is essential to identify factors that mediate the control mechanisms of food preference (that affect food selection and energy metabolism) to increase our understanding of body weight control and the risk of developing chronic diseases, such as obesity [14], which will contribute to the development of individualized treatment strategies [15].

There is limited information regarding the effect of genetics, age, and sex on the sensation of taste and the relationship between taste preference and perception in humans [16], although the oral taste perception system has an essential role in nutrient recognition, determining food preference, and energy balance, which affects eating behavior [17,18,19]. Humans genetically perceive five primary taste attributes (salty, sweet, sour, bitter, and umami) through taste buds located in the oral cavity [14,15,19,20,21].

It is well-known that intake of food is largely regulated by two interacting pathways in the brain. Specifically, one pathway determines food reward and causes the sensation of delight and satisfaction from eating foods or hedonic overeating, whereas the other controls metabolic homeostasis by integrating signals from peripheral tissues with the autonomic nervous system and hormonal system [17,22,23]. Interestingly, the normal function of these two pathways may be affected and decreased by the habitual overconsumption of animal fat. For example, such chronic behavior of overeating in mice increases appetite and a preference for animal fat. Moreover, it may promote the difficulty of obtaining delight and satisfaction after eating such foods [24,25].

High-fat foods are an indispensable source of energy and include lipid-soluble vitamins and essential fatty acids, such as vitamin A [26]. Due to the rise in obesity and its related diseases, the taste of fat and its regulatory mechanisms have been thoroughly researched. The ingestion of dietary oils has a powerful reward effect on the brains of mice [27,28,29]. Thus, the attraction to dietary oils is so powerful that mice that are provided with free access to lipids as a dietary option become obese at a fast rate [26,30]. The relationship between overconsumption of fat and obesity are apparent, but this phenomenon is observed, not only in mice, but also in humans [31,32]. In fact, the consumption of fatty foods is primarily associated with body weight control [15,33,34,35]. Although human individuals enjoy and prefer the taste of fatty foods, they are not able to clearly perceive the taste of fat [15,36].

In addition to its texture-based sensation in the oral cavity, fat has recently been shown to elicit gustatory cues [17]. Accordingly, the taste of free fatty acids (degradation products of triacylglycerides), but not triacylglycerides themselves, may reflect a new primary tastant [36,37,38]. These results suggest the existence of a mechanism through which dietary fat that is sensed in the oral cavity is involved in and mediates fat preference and eating habits.

In this context, removing the gustatory lipid receptor, CD36, results in a considerable decrease in fat preference in mice [39]. This implies that an inadequately regulated fat preference with an impaired sense of taste for fat could exhibit effects on human body weight and body mass index by influencing behavior associated with food intake. Therefore, identifying the factors and underlying mechanisms that mediate fatty food preference, which may include oral fat sensitivity, and determining their relationship with human body weight control will be valuable.

Obesity is not only associated with environmental factors but also strong genetic factors [40,41]. Of the genetic factors, polymorphisms in genes (including single nucleotide polymorphism (SNP)—the most common type of genetic variation) involved in the functional regulation of catecholamine are related to obesity, because catecholamines contribute to energy expenditure and lipolysis [41,42]. In fact, since adrenergic receptors regulate lipid mobilization and energy expenditure, we previously established a relationship between the beta 3-adrenergic receptor (*ADRB3*) and taste preference. The results indicated that a polymorphism in *ADRB3* (Trp64Arg) may be associated with fat preference [15].

Catecholamines and their corresponding receptors, including *ADRB2*, play a major role in regulating energy expenditure [41]. Similar to *ADRB3*, the beta 2-adrenergic receptor (*ADRB2*) is a G-protein-coupled receptor family member, which is made up of seven transmembrane domain-containing proteins. *ADRB2* is composed of a single 413-amino acid residue-long peptide chain. This receptor is encoded by a single intronless gene, which is found on the distal section of the long arm of chromosome 5 (5q32–q34). Although *ADRB2* is expressed in bronchial smooth muscle cells, cardiomyocytes, and vascular smooth muscle cells, it is also expressed in fat cells [41,43]. *ADRB2* regulates the catecholamine-induced adenylate cyclase activation via the action of G proteins [42,43]. The signaling cascade downstream of the receptor also plays an important role in metabolic diseases, such as insulin resistance [44,45].

Polymorphisms in the *ADRB2* gene may be potential genetic factors that explain, in part, the process of human obesity and related traits [44,46]. Various polymorphisms have been detected in the coding region of the *ADRB2* gene. Of these, the Gly16Arg polymorphism of *ADRB2* is a glycine/arginine change at codon 16. A SNP with a G/C missense mutation in the 46th position of the *ADRB2* gene, from GGA to CGA in the *ADRB2* protein (reference SNP number: rs1042713) and the 16th amino acid (Gly) is located in the N-terminal extracellular domain of the *ADRB2* receptor [43]. The genetic polymorphism of *ADRB2* is functionally relevant and thus considered important. Interestingly, this polymorphism may alter the cellular trafficking of *ADRB2* itself and receptor desensitization [46,47,48,49] in addition to the receptor’s agonist affinity [41]. In fact, in vitro ligand binding assays showed that after 24 h of exposure to a beta agonist, isoproterenol, the *ADRB2* isoform with Arg16 underwent increased downregulation as compared with the *ADRB2* isoform with Gly16. This finding was consistent with the observed diminished cyclic adenosine monophosphate response to subsequent isoproterenol treatment after prolonged isoproterenol treatment [50]. Screening studies have demonstrated that the Gly16Arg polymorphism is present in most populations worldwide [51,52]; however, the allele frequencies of this polymorphism differ among ethnic groups. For example, the allele frequencies of the Arg16 allele were 35.1%, 57.5%, and 61% in Caucasian, Asians, and African-Americans, respectively [51,53]. In addition, the allele frequency of the Arg16 was nearly 50% in the healthy Japanese population [52,54].

With respect to the genetic relationship between this *ADRB2* polymorphism and obesity-related metabolic disorders, studies have shown conflicting results and the underlying mechanisms are still unclear. In some studies, the Gly16Arg polymorphism in *ADRB2* was associated with obesity and body mass index (BMI), but the association was not found in others [42,46]. Here, we conducted a study to determine the relationship between the *ADRB2* Gly16Arg (G/C) polymorphism and fatty food preference, which may be associated with the amount of energy intake, because of the essential role of this receptor in fat metabolism. Thus, we examined the effect of the *ADRB2* Gly16Arg (G/C) substitution on food preferences using data acquired from self-reporting questionnaires and genotyping of young and healthy Japanese adults (*n* = 52).

## 2. Materials and Methods

### 2.1. Study Subjects

Inclusion criteria were for young Japanese adult participants (20 to 35 years of age) that were non-smoking and non-medicated, who were healthy when they participated in the testing. Enrollment was also restricted to individuals who achieved a normal saliva flow rate of 1.0 mL/min and 0.1 mL/min during gum-chewing and resting states, respectively [55]. Race and age were restricted to recruit a homogenous group of subjects regarding these factors and to diminish the effect of these factors on preferences of food and other health conditions. Exclusion criteria included smoking and medicated participants because medications and smoking can affect the normal physiological functions of both the central nervous and the stomatognathic systems, including the taste-sensing system [4]. The subjects whose saliva flow rate did not reach a normal level were also excluded because decreased salivary flow may impair physiologic oral functions, such as altered taste perception (which should be normal to evaluate preferences of food), difficulties with mastication, speech, and swallowing, in addition to other clinical oral disorders, such as dental caries [56]. A total of 70 young adults were included in the study through open recruitment. Eighteen were excluded from subsequent data analysis because they failed to complete all of the tests or did not meet the inclusion criteria. The remaining data collected from the 52 young adults were analyzed.

### 2.2. Outline of Study

Participants attending the laboratory at the Tohoku University Graduate School of Dentistry filled out a self-reporting questionnaire on the condition of their health, lifestyles, and eating habits, including various preferences of taste. The participants took part in tests including genotyping of *ADRB2* Gly16Arg (G/C) polymorphisms, a test for saliva flow rate for the first 5 min during resting and mastication to evaluate salivation ability, and the evaluation of basic characteristics for each subject. The weight, height, body fat percentage (the total fat mass divided by total body mass, multiplied by 100), and body mass index (BMI) of each subject were measured with body composition monitors (Inner Scan 50 or BC-314; Tanita, Tokyo, Japan) and a handcrafted stadiometer.

### 2.3. Extraction of DNA and Genotyping of the ADRB2 Polymorphism

The genotype of the *ADRB2* Gly16Arg (G/C) polymorphism was examined by extracting and analyzing the DNA from each participant enrolled in the study. In detail, to clarify the genotypes (GG, GC, or CC) of the *ADRB2* Gly16Arg (G/C) polymorphism, a buccal swab from each participant was acquired with a sterile cotton swab (Tomy Works, Sakai, Japan). Genotyping was outsourced to EBS (Hiroshima, Japan) as previously described [15]. Genomic DNA harvested from each subject was extracted from the swab using the KingFisher Flex Purification System (ThermoFisher Scientific, Paisley, UK) and the MagMAX DNA Multi-Sample Ultra Kit (ThermoFisher Scientific) in accordance with the manufacturer’s guidelines. The genotype was determined by polymerase chain reaction (PCR) using the confronting two-pair primer (PCR-CTPP) method and the KAPA2G Robust PCR Kit (Kapa Biosystems, Wilmington, MA, USA) according to the manufacturer’s guidelines.

### 2.4. Determination of Saliva Flow Rate during Resting and Mastication

Salivation in each participant, in terms of an average saliva flow rate, was determined as previously described [55]. The salivation volume during resting and masticating states was measured for 5 min. We provided an odorless and tasteless chewing gum for saliva collection (Checkbuf salivary gum, Horiba, Kyoto, Japan) to clarify the average whole saliva flow rate that was stimulated with chewing. During the measurement period of the above test, with or without the chewing gum, the participants were requested to expel whole saliva from their mouth into a prepared collection tube at various time points. Participants were also requested to refrain from eating for 2 h before testing. They were also asked to sit in an upright chair for the test and to rinse their mouths with distilled water prior to each test.

### 2.5. Determination of Taste Preference

To evaluate the relationship between the *ADRB2* Gly16Arg (G/C) polymorphism and self-recognition of primary taste preference (salty, sour, sweet, and bitter), we used self-reporting questionnaires. In detail, primary taste preferences were collected using a self-reporting questionnaire that was based on questionnaires of previous studies [15,36,57]. The subjects were asked to answer the ratings of tastants (salty, sour, sweet, or bitter foods) on a scale of 1–5 as follows: scale 5, like very much; scale 4, like moderately; scale 3, neither like nor dislike; scale 2, dislike moderately; scale 1, dislike very much. Six sweet foods described in the questionnaire were classified into two subgroups according to the Standard Tables of Food Composition in Japan 2020 (http://fooddb.mext.go.jp/ (accessed on 13 December 2021)) that shows the fat content contained in each food as follows: (a) low-fat sweet foods (yokan (azuki-bean jelly), manju (a Japanese traditional cake, such as a steamed bean-jam bun), and candy with fat contents of 0.2–0.3 g/100 g, 0.2–3.1 g/100 g, and 0 g/100 g, respectively), and (b) high-fat sweet foods (ice cream, chocolate, and strawberry sponge cake (Japanese-style shortcake)) with fat contents of 2.0–13.6 g/100 g, 34.1–40.4 g/100 g, and 14.7 g/100 g, respectively. The subjects were also asked to rate their degree of greasy (high-fat) food preference on a scale of 1–4, which was previously used in a study by Asano and Watanabe [15,36].

### 2.6. Statistical Analysis

Statistical analyses were conducted using SigmaPlot 14.5 (Systat Software, San Jose, CA, USA), SPSS statistics, version 22.0 (IBM, Armonk, NY, USA), or Excel (Microsoft, Redmond, WA, USA). Differences between group means were identified using appropriate tests as indicated. The relation of age and BMI with each type of food preference was assessed via multiple regression analysis. The allelic and phenotypic frequencies were calculated, and then the Hardy–Weinberg equilibrium was tested for the polymorphism. The chi-square test was employed to compare the frequencies. In each test, *p*-values < 0.05 were defined statistically significant.

## 3. Results

### 3.1. General Characteristics of the Population

Table 1 shows the general characteristics of the study subjects and data for 52 (*n* = 26 male; *n* = 26 female) adults (mean age: 23.4 ± 3.1 years; range: 20 to 35) were analyzed. BMIs ranged from 17.1 to 34.4 kg/m^2^. No significant difference was evident in the average BMI between male and female subjects. Regarding salivation potential, all subjects enrolled in the present study (*n* = 52) exhibited a normal saliva flow rate that was greater than 0.1 mL/min and more than 1.0 mL/min during the resting and masticating states, respectively.

### 3.2. Comparison of Primary Taste Preference Degrees between the Two Sex Subgroups

**Figure 1:**
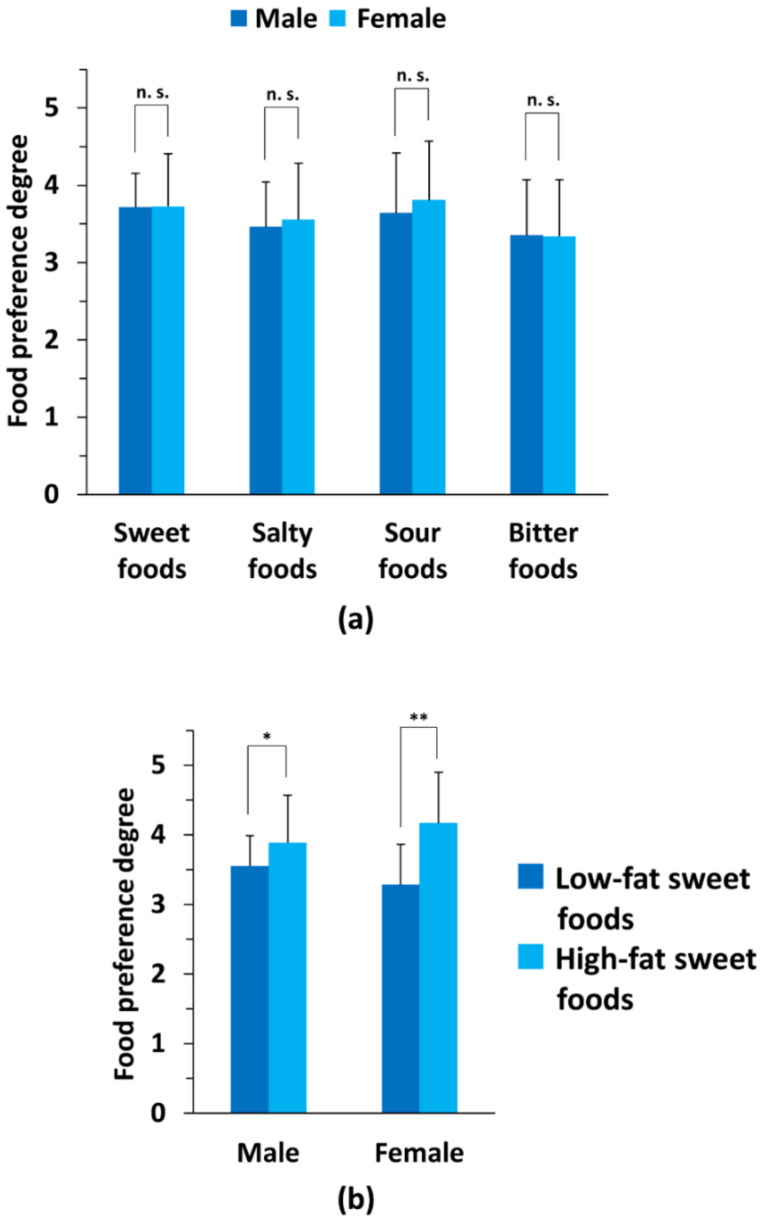
Comparison of primary taste preference degrees between the two sex subgroups. Using a self-reporting questionnaire to evaluate preferences for primary tastes, the subjects rated their degree of taste preference for a variety of tastants including salty, sour, sweet, and bitter on a scale of 1–5 as shown in Table 2, and as follows: scale 5, like very much; scale 4, like moderately; scale 3, neither like nor dislike; scale 2, dislike moderately; scale 1, dislike very much. (**a**) Comparison of the average taste preference between male and female subgroups. The summarized preferences for each primary food taste were compared between men and women (*n* = 52). No significant differences in average taste preferences between males (*n* = 26) and females (*n* = 26) were observed based on Welch’s *t*-test for each tastant. (**b**) Comparison of average taste preference between low-fat and high-fat sweet foods in each sex subgroup. Significant differences were detected between the average taste preference degrees for low-fat and high-fat sweet foods in the female subgroup, but not in the male subgroup. Statistical differences between high-and low-fat sweet food groups were determined using Welch’s *t*-test. For (**a**,**b**), * *p* < 0.05; ** *p* < 0.01; n.s., not significant.

To clarify the relationship between sex differences and primary taste preferences (salty, sour, sweet, and bitter), we compared the male and female subgroups with data acquired from self-reporting questionnaires. As shown in Table 2, the preference extent of each tastant (such as chocolate, salted kelp, grapefruit, and green pepper) was not significantly different between the two sex subgroups (*p* > 0.05). In addition, the average degree of preference for each primary tastant was also not significantly different between the two subgroups (*p* > 0.05; Figure 1a). Next, the six sweet foods listed in the questionnaire were classified into two subgroups and evaluated for average degree of preference for high-fat and low-fat sweet foods (see Materials and Methods for detail). Interestingly, a statistical difference between the two subgroups of sweet foods was observed in both male and female subjects. (Figure 1b).

### 3.3. Genotype Frequencies of ADRB2 Gly16Arg (G/C) Polymorphism in Male and Female Subjects

As shown in Table 3, of the 26 male subjects, seven (26.9%) had the GG genotype, fifteen (57.7%) had the GC genotype, and four (15.4%) had the CC genotype. Of the 26 female subjects, six (23.1%) had the GG genotype, thirteen (50.0%) had the GC genotype, and seven (26.9%) had the CC genotype. In both sexes, no significant difference was observed between the assessed genotype frequencies and the frequencies predicted by the Hardy–Weinberg equilibrium (*p* > 0.05; Table 3). Next, we classified each sex subgroup of subjects into three groups as follows: GG genotype (*ADRB2* GG subgroup, *n* = 7 in male subjects; *n* = 6 in female subjects), GC genotype (*ADRB2* GC subgroup, *n* = 15 in male subjects; *n* = 13 in female subjects), and CC genotype (*ADRB2* CC subgroup, *n* = 4 in male subjects; *n* = 7 in female subjects). To investigate the effect of the *ADRB2* Gly16Arg (G/C) substitution on body fat and BMI, we compared the average values among the three subgroups in male and female subjects. As indicated in Table S1, no significant difference was detected in average BMI or body fat among the *ADRB2* GG, GC, and CC subgroups (*p* > 0.05) for either sex.

### 3.4. Comparison of Primary Taste Preference Degrees between Genotype Subgroups in Male and Female Subjects

**Figure 2:**
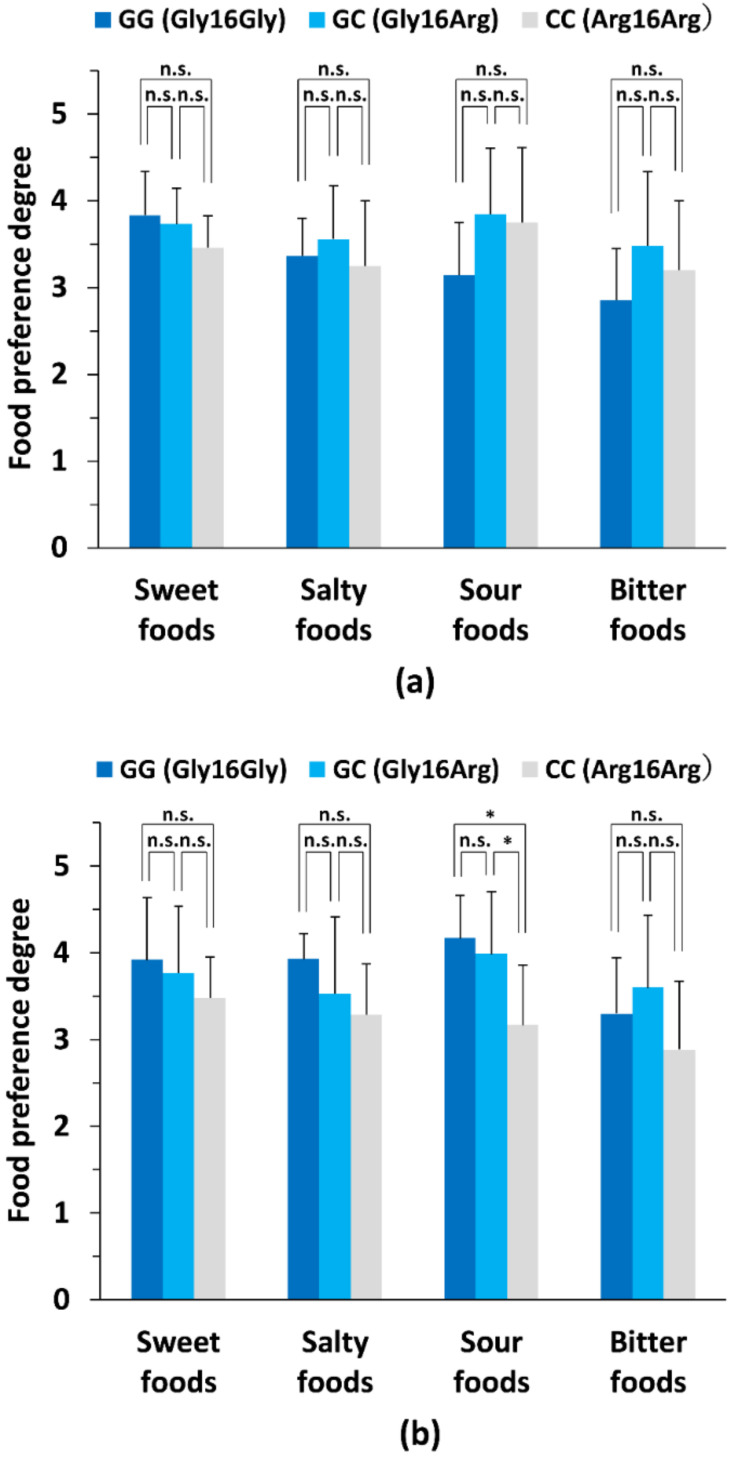
Comparison of primary taste preference degrees between genotype subgroups in male and female subjects. In the self-reporting questionnaire, to determine preferences for primary tastes, subjects rated their degree of taste preferences for a variety of tastants including salty, sour, sweet, and bitter on a scale of 1–5 as presented in Table 4 and Table 5 as follows: scale 5, like very much; scale 4, like moderately; scale 3, neither like nor dislike; scale 2, dislike moderately; scale 1, dislike very much. The average preference degrees for each primary food taste were compared among the indicated genotype subgroups. (**a**) Comparison of primary taste preference degrees between the genotype subgroups in male subjects. For the comparison of average preferences of taste among the genotype subgroups (GG (Gly16Gly) genotype of *ADRB2* (*ADRB2* GG subgroup), GC (Gly16Arg) genotype of *ADRB2* (*ADRB2* GC subgroup), and CC (Gly16Arg) genotype of *ADRB2* (*ADRB2* CC subgroup); *n* = 26), significant differences were found in average taste preference extents among the *ADRB2* GG subgroup (*n* = 7), the *ADRB2* GC subgroup (*n* = 15), and the *ADRB2* CC subgroup (*n* = 4) genotype subgroups in male subjects, identified by performing a one-way analysis of variance followed by the Holm–Sidak test for each indicated tastant. (**b**) Comparison of primary preference degrees between genotype subgroups in female subjects. For the comparison of average taste preferences among the genotype subgroups (*ADRB2* GG subgroup, *ADRB2* GC subgroup, and *ADRB2* CC subgroup; *n* = 26), significant differences were found in average taste preference degrees among the *ADRB2* GG subgroup (*n* = 6), the *ADRB2* GC subgroup (*n* = 13), and the *ADRB2* CC subgroup (*n* = 7) genotype subgroups in female subjects, identified by performing a one-way analysis of variance followed by the Holm–Sidak test for each indicated tastant. For (**a**,**b**), * *p* < 0.05; n.s., not significant.

Among the *ADRB2* genotypes, there were significant differences in preference for pickled Japanese plum (genotype: GG vs. GC, *p* < 0.05) and grapefruit (genotype: GG vs. GC, *p* < 0.05; GG vs. CC, *p* < 0.05) in male subjects (Table 4). In addition, as indicated in Table 5, among the *ADRB2* genotypes, there were significant differences in preference for pickled vegetables (genotype: GG vs. CC, *p* < 0.05), grapefruit (genotype: GC vs. CC, *p* < 0.05), and coffee (genotype: GG vs. GC, *p* < 0.01; GC vs. CC, *p* < 0.05) in female subjects. However, based on the summarized preference degree for each primary tastant (sweet, salty, sour, and bitter) in each genotype subgroup of the male subjects, no significant difference was observed in preferences for the foods of each taste (*p* > 0.05) as shown in Figure 2a. In contrast, as indicated in Figure 2b, for female subjects, a significant difference was observed in the preference for sour-tasting food (genotype: GG vs. CC, *p* < 0.05; GC vs. CC, *p* < 0.05).

### 3.5. Comparison of the Degree of Taste Preference between Low-Fat and High-Fat Sweet Tastes in Each Genotype Subgroup for Male and Female Subjects

**Figure 3:**
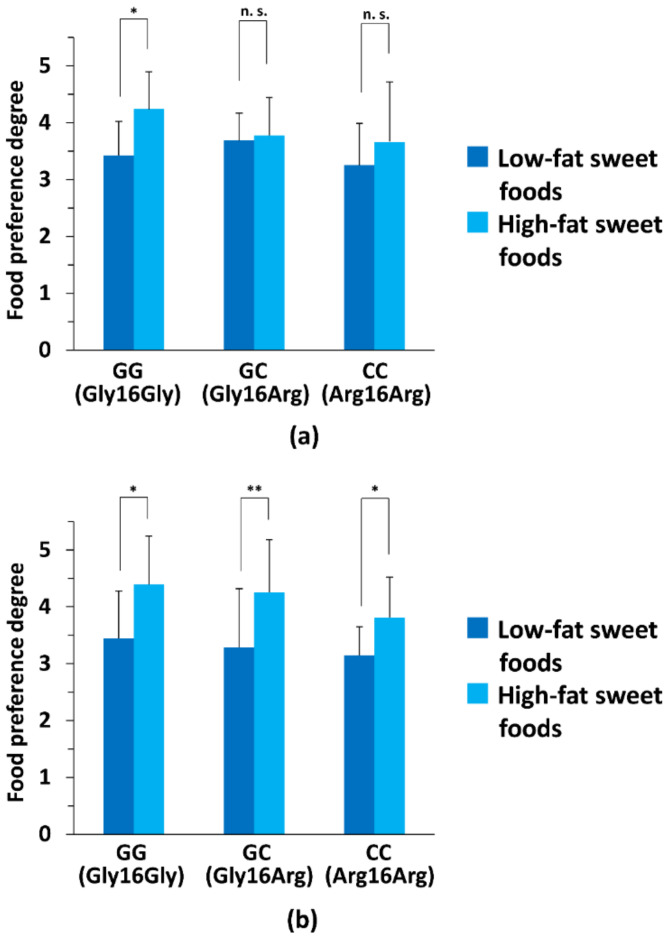
Comparison of the degrees of taste preference between high- and low-fat sweet tastes in each genotype subgroup for male and female subjects. The six sweet foods listed in the self-reporting questionnaire were classified into two subgroups (low-fat sweet foods (yokan, manju, and candy) and high-fat sweet foods (ice cream, chocolate, and strawberry sponge cake)) according to general fat content. (**a**) Comparison of taste preference degrees between low-fat and high-fat sweet tastes in each genotype subgroup for the male subjects. Significant differences were determined between taste preference degrees for low-fat and high-fat sweet foods in each genotype subgroup (subjects with the GG (Gly16Gly) genotype of *ADRB2* (*ADRB2* GG subgroup, *n* = 7), with the GC (Gly16Arg) genotype of *ADRB2* (*ADRB2* GC subgroup, *n* = 15), and with the CC Gly16Arg] genotype of *ADRB2* (*ADRB2* CC subgroup, *n* = 4) of male subjects (*n* = 26)). Statistical differences between low-fat and high-fat sweet food groups were determined using a Welch’s *t*-test. (**b**) Comparison of taste preference extents between low-fat and high-fat sweet tastes in each genotype subgroup for the female subjects. Significant differences were determined between summarized taste preference degrees for low-fat and high-fat sweet foods for each genotype subgroup (*ADRB2* GG subgroup, *n* = 6; *ADRB2* GC subgroup, *n* = 13; *ADRB2* CC subgroup, *n* = 7) of female subjects (*n* = 26). Statistical differences between low-fat and high-fat sweet food groups were determined using a Welch’s *t*-test. For (**a**,**b**), * *p* < 0.05; ** *p* < 0.01; n.s., not significant.

Regarding the six sweet foods included in the questionnaire, the summarized degree of taste preference for the three high-fat sweet foods in male subjects was significantly higher compared with that of the three low-fat sweet foods in the *ADRB2* GG subgroup only, but not in the other genotype subgroups of *ADRB2* (*p* < 0.05; Figure 3a). In female subjects, however, the average extent of taste preference for the three high-fat sweet foods was significantly higher compared with that of the three low-fat sweet foods in all genotype subgroups (*ADRB2* GG, GC, and CC subgroups), but not in the *ADRB2* CC subgroup (*p* < 0.05; Figure 3b).

### 3.6. Comparison of Greasy Food Preference Degrees among Genotype Subgroups in Male and Female Subjects

**Figure 4:**
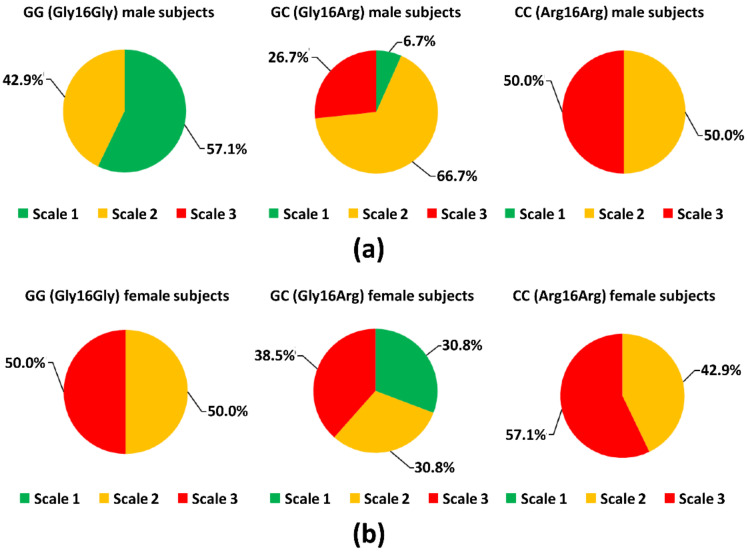

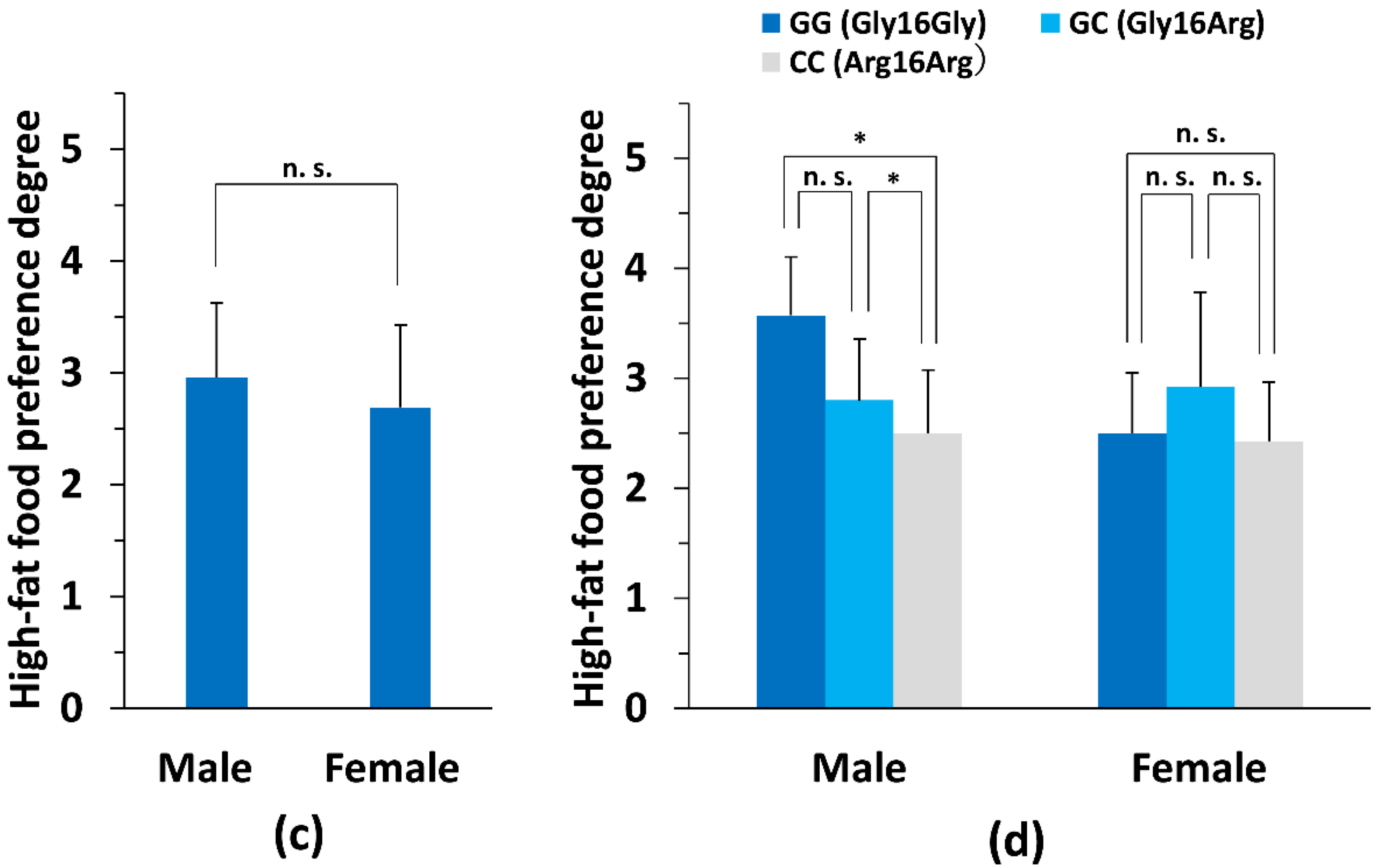
Comparison of greasy food preference degrees among genotype subgroups in male and female subjects. In the self-reporting questionnaire for preference of taste, participants rated their preference for greasy foods on a scale of 1–4 as shown in Table 6 as follows: scale 4, dislike very much (score: 1); scale 3, dislike moderately (score: 2); scale 2, like moderately (score: 3); scale 1, like very much (score: 4). The percentage of male or female subjects who chose each value and the average extents of preference were compared among the indicated genotype subgroups. (**a**) Pie chart indicating the percentage of subjects who chose each number (scale: 1–4) in participants from each genotype subgroup (subjects with GG (Gly16Gly) genotype of *ADRB2* (*ADRB2* GG subgroup, *n* = 7), with GC (Gly16Arg) genotype of *ADRB2* (*ADRB2* GC subgroup, *n* = 15), and with CC (Gly16Arg) genotype of *ADRB2* (*ADRB2* CC subgroup, *n* = 4) for the male subjects (*n* = 26)). No participants selected 4 in the self-reporting questionnaire. (**b**) Pie chart indicating the percentage of subjects who chose each number (scale: 1–4) for the participants with each genotype subgroup (GG (Gly16Gly) genotype of *ADRB2* (*ADRB2* GG subgroup, *n* = 6), with GC (Gly16Arg) genotype of *ADRB2* (*ADRB2* GC subgroup, *n* = 13), and with CC (Gly16Arg) genotype of *ADRB2* (*ADRB2* CC subgroup, *n* = 7) for the female subjects (*n* = 26). No subject chose 4 in the self-reporting questionnaire. (**c**) Comparison of average greasy food preference degree between male and female subgroups (*n* = 52). Statistical differences between male and female subgroups were detected using a Welch’s *t*-test. (**d**) Comparison of average greasy food preference degree among genotype subgroups (*ADRB2* GG subgroup, *ADRB2* GC subgroup, and *ADRB2* CC subgroup) for each sex subgroup. For comparison of the average greasy food preference among the genotype subgroups representing each sex, significant differences in average taste preference extents among the subgroups were detected by conducting a one-way analysis of variance followed by the Holm–Sidak test. For (**c**,**d**), * *p* < 0.05; n.s., not significant.

To determine the relationship between the *ADRB2* Gly16Arg (G/C) polymorphism and the self-recognition of taste preferences for greasy (high-fat) foods among the subjects, we focused on a specific question included in the self-reporting questionnaire (Table 6), which was also included in previous studies [15,36]. As shown in Table 6 and Figure 4a,b, we observed that the preference degree for greasy foods largely differed among the study subjects, although no subject disliked greasy foods highly. However, the percentage of subjects who liked greasy foods highly tended to be significantly larger in the *ADRB2* GG subgroup compared with the other *ADRB2* genotype subgroups for the male subjects (Table 6 and Figure 4a). In contrast, this tendency was not observed in female subjects (Table 6 and Figure 4b). Moreover, although we observed that there were no significant differences in the average preference degree for greasy foods between male and female subjects as indicated in Figure 4c, the average preference degree for greasy foods of the *ADRB2* GG subgroup was significantly higher compared with that of the *ADRB2* CC subgroup in male subjects, but not in female subjects (*p* < 0.01; Figure 4d).

### 3.7. Multiple Linear Regression Anaysis Using Age and BMI in Male aad Female Subjects

Finally, to access the effect of age and BMI on each type of food preference evaluated in the present study (sweet food, salty food, sour food, bitter food, and high-fat food preference), multiple linear regression analysis was performed using the data acquired from the questionnaire in the present study. No significant association was observed between each type of food preference and age or BMI in both male and female subjects (Table S2).

## 4. Discussion

In the current study (all subjects being Japanese), the frequency in male and female subjects was 44.2% and 51.9%, respectively, which was consistent with the previous findings on the *ADRB2* Arg16 allelic frequency (see Introduction for details). Additionally, the observed genotype frequencies of the Gly16Arg polymorphism of the *ADRB2* gene in both male and female subjects were in accordance with the Hardy–Weinberg equilibrium, suggesting that the observed genotype frequencies in both males and females represent those of the entire population. Thus, we analyzed the effect of the Gly16Arg (G/C) substitution of *ADRB2* on preferences of taste with data from both male and female subjects and found that the preference for sour food was significantly higher in the *ADRB2* GG group than that in the *ADRB2* CC group, but only for female subjects. When sweet foods were classified into low- and high-fat subgroups, the preference for high-fat sweet foods was higher than that for low-fat sweet foods in the *ADRB2* GG group for both male and female subjects. Furthermore, we also found that the degree of preference for high-fat foods was significantly higher in the *ADRB2* GG group than that in the *ADRB2* CC group, but only for male subjects.

*ADRB2* mediates insulin resistance, cardiorespiratory fitness, and obesity [58]. It has a dominant effect as a major lipolytic receptor in human fat cells [41,59]. The Gly16Arg (G/C) substitution of *ADRB2*, which appears to be of significant importance to *ADRB2* function, at least in fat cells, but less so for obesity, may induce a significant decrease in agonist affinity for *ADRB2* and/or a significant increase in *ADRB2* downregulation [41,50]. For example, the polymorphism of *ADRB2* may also be associated with altered glucose metabolism [58]. However, to our knowledge, no study has analyzed the potential effect of this polymorphism on the self-recognition of basic and high-fat taste preferences. Thus, we characterized the relationship between this polymorphism and the self-recognition of various taste food preferences in healthy Japanese young adults.

This is the first study to characterize significant associations between the *ADRB2* Gly16Alg (G/C) polymorphism and taste preferences in healthy and young male and female subjects. As described above, our results clearly show that there is a specific association of the *ADRB2* Gly16Arg (G/C) substitution with average degree of sour food preference in females, but not in males, as well as the average extent of high-fat preference in males, but not in females. Preferences for other basic tastes (salty, sweet, and bitter tastes) were not apparently changed by this alteration. These results suggest that the difference in the degree of affinity of the agonist to *ADRB2* and/or in cellular trafficking inducing downregulation of *ADRB2* itself (this event might be involved in determining the genotype-dependent lipolytic activity of *ADRB2* in fat cells and changing the energy expenditure of the human body) between the GG and CC genotypes for this polymorphism may subsequently induce alterations in taste preferences, rather than that of BMI, at least in a complex and sex-dependent manner, despite an unknown underlying mechanism. In particular, it is unclear why there were no significant differences in fat preference among the three genotype subgroups of the *ADRB2* polymorphism in females, but not males.

Previous studies indicated that fat preference can be affected by female hormones in mice and rodents [17,26]. Thus, there is the possibility that the effect of female hormones in female subjects may also predominantly contribute to the regulation of fat preference in humans rather than this polymorphism. This suggests the possibility that the effects of female hormones mask or weaken the effect of the genotype-dependent difference in *ADRB2* activity and its location in cells (that may subsequently affect the energy expenditure of the human body) on fat preference to some extent in female, but not male subjects.

Interestingly, as described above, we also found that in male but not female subgroups, similar to the observed sex-dependent difference in fat preference, the average high-fat sweet food preference was significantly higher than the low-fat sweet preference for the GG genotype, but not the CC genotype only in male subjects. In this context, it should be noted that foods with high-fat sweet tastes can cause a hyperpalatable sensation in the human brain [60,61]. Therefore, such hyperpalatable foods may have a special effect on human brain function compared with other foods, including only fatty foods. They may be able to differentially affect the regulatory mechanism of taste preferences in carriers of the GG genotype in some specific manner, although the precise mechanism is unknown and further studies are required.

In addition, with respect to the female-specific upregulation of sour-taste food preferences in the subjects with a GG genotype, we should note the following two points: (i) the sour-taste quality can occasionally be a sign of citric acid, which is included in a variety of sour foods, such as grapefruit, oranges, and lemons. In addition, citric acid is a pivotal player in the tricarboxylic acid cycle in mitochondria, which is a sequence in a metabolic pathway that mediates the chemical conversion of carbohydrates, proteins, and fats into water and carbon dioxide to produce energy [62]; and (ii) the subjects with the GG genotype may require higher amounts of energy per day because they have significantly higher *ADRB2* receptor activity (that generally leads to an increase of energy expenditure), compared with those containing the CC genotype [41]. Accordingly, we cannot refute the possibility that the upregulation of the sour preference in female subjects with the GG genotype may be associated with the enhanced energy expenditure of the human body for GG genotype females.

The genetically enhanced sour and fat food preferences described above, which we found in the individuals with the GG genotype compared with the CC genotype, could be an advantage and may be useful. The subjects with the GG genotype may be able to select high-fat foods more efficiently among various foods, which may compensate for the upregulated *ADRB2* receptor activity and/or decreased downregulation of the receptor in subjects with GG genotype. Thus, the observed enhancement of fat preference that was not apparently linked to obesity may be related not only to the increased caloric intake, but also to obtaining satisfaction for the sufficient amount of food they consume.

Regardless of the above working hypotheses, the precise mechanism through which the *ADRB2* Gly16Arg (G/C) substitution affects fat and sour taste preferences was not clarified in the present study. With respect to the altered fat and sour preferences, it should be noted that *ADRB2* is an indispensable mediator of fat metabolism [58], which is partially mediated by the TCA cycle in the mitochondria [62]. Thus, altered *ADRB2* receptor activity and its distribution in cells with this polymorphism, followed by changes in fat metabolism in brown and white adipose tissue, may influence the principal pathways that control intake of food in the brain, such as pathways that regulate (i) metabolic homeostasis and (ii) food reward via an unrevealed mechanism. Therefore, further studies on the effects of gene mutations in *ADRB2* on food preferences with respect to brain function on the metabolic regulation of the human body will contribute to reveal the precise mechanism through which the *ADRB2* Gly16Arg variant affects preferences of sour and fat.

In relation to the effect of the *ADRB2* Gly16Arg (G/C) substitution on BMI, our results in addition to several other reports, failed to demonstrate a significant change in BMI associated with the Gly16Arg substitution [41,42,44,46], in spite of the fact that both changes in basal metabolic expenditure and fat preference (the latter first shown in the current study for the *ADRB2* Gly16Arg polymorphism) can affect the homeostasis of human body weight [26,63,64]. Although the precise reason is currently unknown, there are some possibilities as to why no differences were observed for this polymorphism as follows. First, a combination of related factors may lead to obesity. Therefore, increased energy expenditure followed by exercise may compensate for the differences in fat preference or metabolic expenditure induced by the genetic alteration of *ADRB2*. In addition, in this context, it should be noted that long-term moderate exercise training itself can significantly increase the gene expression level of *ADRB2* in humans [65]. Second, the age of the participants was restricted to young adults (in their 20s and 30s). Thus, basal metabolic expenditure was relatively high compared with that of older adults. In fact, the percentage of obese subjects (i.e., whose BMI was 30 or above) was only 1.923% in our present study. Thus, this may compensate for the difference in fat preference or metabolic expenditure resulting from the Gly16Arg substitution to some extent.

The following study limitations should be considered. First, the small number of subjects may have resulted in low statistical power; however, this population was sufficient to show an association between preference of taste and the *ADRB2* Gly16Arg (G/C) polymorphism. Second, the self-reporting style of the questionnaire may have resulted in individual biases that may have affected the results. Our research group prepared the questions presented in Tables in the Japanese language. In particular, the participants received and responded to the Japanese language version of the questionnaire, rather than the translated English language version shown in the tables. Thus, it is possible that some bias may have occurred during translation. A third limitation is the effect of sex differences on taste preferences. Studies have demonstrated the influence of hormones on preferences of taste [17,66]. Therefore, to satisfy diverse physiological needs, it is reasonable that males and females exhibit different dietary intakes and storage [17]. However, sex is a related parameter that has been poorly analyzed regarding taste preference [15,16,67,68]. In fact, when we compared male and female subjects, we found a few differences in basic taste preferences in our present study. Thus, we separately analyzed the genotypic data between the male and female subgroups. However, the preference of taste degree toward each food in the present study was averaged, but the transient status was not included. Accordingly, we did not acquire data on the hormone status (including female hormones) in the male and female subjects in the self-reporting questionnaire and did not separate the subjects into subgroups based on the status of hormone or other related variables. Notwithstanding, future studies regarding taste preferences in the context of hormonal status for men and women subjects may have significant value to better understand the relationship of the *ADRB2* Gly16Arg (G/C) polymorphism with taste preferences. Finally, the study subjects were non-smoking, non-medicated, Japanese young adults in good health and may not accurately reflect the entire population.

## 5. Conclusions

In the current study, we focused on the genetic relationship between taste preferences and the Gly16Arg (G/C) polymorphism of *ADRB2* in healthy, young, adult Japanese men and women. We concluded that sour and fat preferences were specifically associated with this polymorphism based on self-recognition, and the manner of association was different between males and females. These findings are essential, especially for the advancement of personalized medicine, as genetically altered preferences for both sour and fatty foods (that includes high-fat sweet foods) may be associated with the promotion of inadequate eating habits, such as overeating. This may contribute, in part, to regulating the risk of diabetes, severe obesity, or other lifestyle-related disorders in humans. Taken together, our results suggest that the *ADRB2* Gly16Arg (G/C) polymorphism may have a significant correlation with the self-recognition of food preferences in healthy young adults via an unknown mechanism. Further research on the relationship between this polymorphism and fatty and sour food preferences will be highly valuable to identify the control mechanisms underlying the preferences and to develop tools for individualized medicine to analyze personal- and sex-dependent differences in sour and fatty preferences. This will contribute to the assessment of *ADRB2*-related functional shifts in sour and fatty taste preferences, which may be used to manage or prevent obesity promoted by multiple causative factors, such as this polymorphism.

## Figures and Tables

**Table 1:** General characteristics of the subjects.

	All (Japanese, *n* = 52)			Male (*n* = 26)			Female (*n* = 26)			Male vs. Female
	Mean	SD	Range	Mean	SD	Range	Mean	SD	Range	*p*-Value ^#^
Age (year)	23.4	3.1	20–35	23.7	4.2	20–35	23.1	2.9	20–33	n.s.
Height (cm)	166.6	8.6	151.1–182.5	173.6	5.9	165.6–182.5	159.7	5.0	151.1–168.4	<0.0001 **
Weight (kg)	59.5	11.3	44.2–105.0	66.3	9.4	49.6–105.0	52.6	5.1	44.2–65.2	<0.0001 **
BMI (kg/m^2^)	21.3	2.9	17.1–34.4	22.0	2.7	17.1–34.4	20.6	2.0	17.8–26.6	n.s.
Body fat (%)	21.9	8.0	6.9–36.0	15.6	5.4	6.9–33.4	28.1	3.7	21.6–36.0	<0.0001 **

Data are shown as the means with standard deviation (SD). BMI, body mass index. ^#^ Statistical difference between male and female subjects using a Welch’s *t*-test. ** *p* < 0.01; n.s., not significant.

**Table 2:** Preferences of food in male and female subjects.

		All (Japanese, *n* = 52)		Male (*n* = 26)		Female (*n* = 26)		Male vs. Female
		Mean	SD	Mean	SD	Mean	SD	*p*-Value ^#^
Sweet food	Azuki-bean jelly (Yokan)	3.40	1.12	3.58	0.81	3.23	1.37	n.s.
	Steamed bean-jam bun (Manju)	3.63	0.97	3.69	0.88	3.58	1.06	n.s.
	Candy	3.21	1.00	3.38	0.85	3.04	1.11	n.s.
	Ice cream	4.27	0.91	4.23	0.76	4.31	1.05	n.s.
	Chocolate	4.02	1.00	3.77	0.95	4.27	1.00	n.s.
	Strawberry sponge cake (Japanese-style shortcake)	3.79	1.07	3.65	0.98	3.92	1.16	n.s.
Salty food	Potato chips	3.88	1.08	4.04	0.82	3.73	1.28	n.s.
	Salted squids (Ika shiokawa)	2.90	1.35	2.96	1.31	2.85	1.41	n.s.
	Salted kelp (Shio Kombu)	3.06	1.23	3.00	1.02	3.12	1.42	n.s.
	Pickled vegetables (Tsukemono)	3.35	1.08	3.15	0.92	3.54	1.21	n.s.
	Salted cod roe (Tarako)	3.48	1.26	3.31	1.12	3.65	1.38	n.s.
	Salted salmon	3.73	0.93	3.77	0.82	3.69	1.05	n.s.
	Miso soup (Misoshiru)	4.15	0.83	4.00	0.80	4.31	0.84	n.s.
Sour food	Japanese orange (Mikan)	4.35	0.95	4.38	0.80	4.31	1.09	n.s.
	Hassaku orange	3.62	1.03	3.46	0.95	3.77	1.11	n.s.
	Pickled Japanese plum (Umeboshi)	3.44	1.27	3.38	1.13	3.50	1.42	n.s.
	Yogurt	4.19	0.86	4.08	0.84	4.31	0.88	n.s.
	Lemon	3.19	1.09	3.12	1.07	3.27	1.12	n.s.
	Grapefruit	3.56	1.19	3.42	1.17	3.69	1.23	n.s.
Bitter food	Celery	2.54	1.24	2.42	1.06	2.65	1.41	n.s.
	Tea	4.37	0.69	4.35	0.63	4.38	0.75	n.s.
	Green pepper	3.38	1.07	3.58	1.03	3.19	1.10	n.s.
	Parsley	2.81	1.24	2.85	1.19	2.77	1.31	n.s.
	Coffee	3.63	1.19	3.58	1.24	3.69	1.16	n.s.

Data are shown as the means with standard deviation (SD); n.s., not significant. ^#^ Statistical difference between male and female subjects using a Welch’s *t*-test.

**Table 3:** Genotype frequencies of *ADRB2* Gly16Arg (G/C) polymorphism in male and female subjects.

SNP	Male (*n* = 26)					Female (*n* = 26)				
	Ratio of Genotypes			Chi-Square Test		Ratio of Genotypes			Chi-Square Test	
*ADRB2*	GG	GC	CC	χ2	*p*-Value ^#^	GG	GC	CC	χ2	*p*-Value
Gly16Arg	(Gly16Gly)	(Gly16Arg)	(Arg16Arg)			(Gly16Gly)	(Gly16Arg)	(Arg16Arg)		
(refSNP ^#^: rs1042713)	*n* = 7 (26.9)	*n* = 15 (57.7)	*n* = 4 (15.4)	0.746	n.s.	*n* = 6 (23.1)	*n* = 13 (50.0)	*n* = 7 (26.9)	<0.001	n.s.

The *n* (value) means number of participants (percentage). The Hardy–Weinberg equilibrium and chi-square test were used to assess whether the observed genotype frequencies were different from the frequencies predicted by the equilibrium. No significant difference was observed between them for the male and female subjects. Replacement of the guanine (G) in the 46th position to cytosine (C) changes the 16th amino acid glycine (Gly) into an arginine (Arg) residue in the N-terminal extracellular domain of the human beta 2-adrenergic receptor (*ADRB2*). n.s., not significant. ^#^ The reference SNP number (RefSNP) is provided where available in the public database. SNP, single nucleotide polymorphism.

**Table 4:** Food preference in male subjects with the GG (Gly16Gly), GC (Gly16Arg), and CC (Arg16Arg) genotypes of *ADRB2*.

		GG (Gly16Gly) (*n* = 7)		GC (Gly16Arg) (*n* = 15)		CC (Arg16Arg) (*n* = 4)		GG vs. GC	GG vs. CC	GC vs. CC
		Mean	SD	Mean	SD	Mean	SD	*p*-Value ^#^	*p*-Value ^#^	*p*-Value ^#^
Sweet food	Azuki-bean jelly (Yokan)	3.43	0.98	3.67	0.72	3.50	1.00	n.s.	n.s.	n.s.
	Steamed bean-jam bun (Manju)	3.29	1.11	3.93	0.70	3.50	1.00	n.s.	n.s.	n.s.
	Candy	3.57	1.13	3.47	0.52	2.75	1.26	n.s.	n.s.	n.s.
	Ice cream	4.57	0.79	4.13	0.74	4.00	0.82	n.s.	n.s.	n.s.
	Chocolate	4.00	0.82	3.73	0.96	3.50	1.29	n.s.	n.s.	n.s.
	Strawberry sponge cake (Japanese style shortcake)	4.14	0.90	3.47	0.92	3.50	1.29	n.s.	n.s.	n.s.
Salty food	Potato chips	4.29	0.76	3.93	0.88	4.00	0.82	n.s.	n.s.	n.s.
	Salted squids (Ika shiokawa)	3.14	1.35	3.20	1.26	1.75	0.96	n.s.	n.s.	n.s.
	Salted kelp (Shio Kombu)	2.86	1.07	3.13	0.99	2.75	1.26	n.s.	n.s.	n.s.
	Pickled vegetables (Tsukemono)	2.86	0.90	3.27	0.80	3.25	1.50	n.s.	n.s.	n.s.
	Salted cod roe (Tarako)	3.43	1.13	3.27	1.22	3.25	0.96	n.s.	n.s.	n.s.
	Salted salmon	3.43	0.53	3.87	0.92	4.00	0.82	n.s.	n.s.	n.s.
	Miso soup (Misoshiru)	3.57	0.79	4.27	0.80	3.75	0.50	n.s.	n.s.	n.s.
Sour food	Japanese orange (Mikan)	4.29	0.76	4.47	0.83	4.25	0.96	n.s.	n.s.	n.s.
	Hassaku orange	3.14	0.90	3.67	0.98	3.25	0.96	n.s.	n.s.	n.s.
	Pickled Japanese plum (Umeboshi)	2.43	0.98	3.73	0.96	3.75	1.26	0.028 *	n.s.	n.s.
	Yogurt	3.86	0.90	4.20	0.86	4.00	0.82	n.s.	n.s.	n.s.
	Lemon	2.71	0.76	3.27	1.03	3.25	1.71	n.s.	n.s.	n.s.
	Grapefruit	2.43	0.98	3.73	1.10	4.00	0.82	0.034 *	0.047 *	n.s.
Bitter food	Celery	2.00	1.15	2.60	1.12	2.50	0.58	n.s.	n.s.	n.s.
	Tea	3.86	0.38	4.53	0.64	4.50	0.58	n.s.	n.s.	n.s.
	Green pepper	2.86	1.07	3.93	0.96	3.50	0.58	n.s.	n.s.	n.s.
	Parsley	2.43	1.13	3.07	1.33	2.75	0.50	n.s.	n.s.	n.s.
	Coffee	3.14	0.69	4.00	1.36	2.75	0.96	n.s.	n.s.	n.s.

Data are shown as the means with standard deviation (SD). ^#^ Statistical difference among the above indicated genotypes of *ADRB2* using a one-way analysis of variance followed by the Holm–Sidak test. * *p* < 0.05; n.s., not significant.

**Table 5:** Food preference in female subjects with the GG (Gly16Gly), GC (Gly16Arg), and CC (Arg16Arg) genotypes of *ADRB2*.

		GG (Gly16Gly) (*n* = 6)		GC (Gly16Arg) (*n* = 13)		CC (Arg16Arg) (*n* = 7)		GG vs. GC	GG vs. CC	GC vs. CC
		Mean	SD	Mean	SD	Mean	SD	*p*-Value ^#^	*p*-Value ^#^	*p*-Value ^#^
Sweet food	Azuki-bean jelly (Yokan)	3.00	1.41	3.46	1.36	3.00	1.00	n.s.	n.s.	n.s.
	Steamed bean-jam bun (Manju)	3.83	0.75	3.62	1.39	3.29	0.49	n.s.	n.s.	n.s.
	Candy	3.50	1.52	2.77	0.93	3.14	1.07	n.s.	n.s.	n.s.
	Ice cream	4.67	0.52	4.23	1.30	4.14	0.90	n.s.	n.s.	n.s.
	Chocolate	4.50	0.84	4.46	0.97	3.71	1.11	n.s.	n.s.	n.s.
	Strawberry sponge cake (Japanese style shortcake)	4.00	1.55	4.08	1.04	3.57	1.13	n.s.	n.s.	n.s.
Salty food	Potato chips	4.00	1.67	3.23	1.17	4.43	0.79	n.s.	n.s.	n.s.
	Salted squids (Ika shiokawa)	3.50	1.22	2.85	1.63	2.29	0.95	n.s.	n.s.	n.s.
	Salted kelp (Shio Kombu)	3.33	1.51	3.38	1.50	2.43	1.13	n.s.	n.s.	n.s.
	Pickled vegetables (Tsukemono)	4.50	0.55	3.46	1.27	2.86	1.07	n.s.	0.038 *	n.s.
	Salted cod roe (Tarako)	4.50	0.84	3.62	1.50	3.00	1.29	n.s.	n.s.	n.s.
	Salted salmon	3.17	0.98	3.85	1.21	3.86	0.69	n.s.	n.s.	n.s.
	Miso soup (Misoshiru)	4.50	0.84	4.31	0.85	4.14	0.90	n.s.	n.s.	n.s.
Sour food	Japanese orange (Mikan)	4.83	0.41	4.31	1.03	3.86	1.46	n.s.	n.s.	n.s.
	Hassaku orange	3.67	0.82	4.08	1.04	3.29	1.38	n.s.	n.s.	n.s.
	Pickled Japanese plum (Umeboshi)	4.00	1.67	3.69	1.32	2.71	1.25	n.s.	n.s.	n.s.
	Yogurt	4.67	0.82	4.38	0.87	3.86	0.90	n.s.	n.s.	n.s.
	Lemon	3.83	0.98	3.38	1.12	2.57	0.98	n.s.	n.s.	n.s.
	Grapefruit	4.00	1.26	4.08	1.12	2.71	0.95	n.s.	n.s.	0.045 *
Bitter food	Celery	3.17	1.60	2.85	1.46	1.86	0.90	n.s.	n.s.	n.s.
	Tea	4.67	0.52	4.46	0.66	4.00	1.00	n.s.	n.s.	n.s.
	Green pepper	3.17	0.75	3.38	1.39	2.86	0.69	n.s.	n.s.	n.s.
	Parsley	2.67	1.51	2.92	1.44	2.57	0.98	n.s.	n.s.	n.s.
	Coffee	2.83	1.33	4.38	0.96	3.14	0.38	0.009 **	n.s.	0.021 *

Data are shown as the means with standard deviation (SD). ^#^ Statistical difference among the above indicated genotypes of *ADRB2* using a one-way analysis of variance followed by the Holm–Sidak test. * *p* < 0.05; ** *p* < 0.01; n.s., not significant.

**Table 6:** The response to a question on greasy food preference in male and female subjects with the GG (Gly16Gly), GC (Gly16Arg), and CC (Arg16Arg) genotypes of *ADRB2*.

Question: Do You Like Greasy Foods?		Genotype: GG (Gly16Gly)				Genotype: GC (Gly16Arg)				Genotype: CC (Arg16Arg)			
		Male (*n* = 7)		Female (*n* = 6)		Male (*n* = 15)		Female (*n* = 13)		Male (*n* = 4)		Female (*n* = 7)	
Scale	Score	*n*	%	*n*	%	*n*	%	*n*	%	*n*	%	*n*	%
1. I like them very much	4	4	13.8	0	0.0	1	8.3	4	13.8	0	0.0	0	0.0
2. I like them moderately	3	3	10.3	3	25.0	10	83.3	4	13.8	2	16.7	3	25.0
3. I dislike them moderately	2	0	0.0	3	25.0	4	33.3	5	17.2	2	16.7	4	33.3
4. I dislike them very much	1	0	0.0	0	0.0	0	0.0	0	0.0	0	0.0	0	0.0

The question included in the self-reporting questionnaire was for determining the greasy food preference extent for each subject.

## Data Availability

The data acquired and analyzed during this study are included in this article. More information is available from the corresponding author on reasonable request.

## Supplementary Materials

The following supporting information can be downloaded at: https://www.mdpi.com/article/10.3390/nu14071430/s1, Table S1: The effects of *ADRB2* Gly16Arg (G/C) polymorphism on BMI and body fat in male and female subjects.; Table S2: Multiple linear regression analysis using age and BMI as dependent variables in male and female subjects.
