# Supplementary material for: Effect of Beta 2-Adrenergic Receptor Gly16Arg Polymorphism on Taste Preferences in Healthy Young Japanese Adults
Source: Nutrients. 2022 Mar 29;14(7):1430. doi: 10.3390/nu14071430 (PMC9003210; doi:10.3390/nu14071430)
Supplement: Supplementary file 1 [file nutrients-14-01430-s001.zip › nutrients-1614714-supplementary.pdf]

Supplementary Information

Table S1. The effects of *ADRB2* Gly16Arg (G/C) polymorphism on BMI and body fat in male and female subjects.

	Male (*n* = 26)									GG vs. GC *P*-value#	GG vs. CC *P*-value#	GC vs. CC *P*-value#
	GG(Gly16Gly) (*n* = 7)			GC(Gly16Arg) (*n* = 15)			CC(Arg16Arg) (*n* = 4)					
	Mean	SD	Range	Mean	SD	Range	Mean	SD	Range			
BMI (kg/m^2)	23.0	5.3	18.1–34.4	21.8	2.9	17.1–28.2	20.7	1.7	18.3–22.3	n.s.	n.s.	n.s.
Body fat (%)	16.2	8.1	9.9–33.4	15.5	5.3	6.9–24.3	13.9	2.4	11.6–17.2	n.s.	n.s.	n.s.

	Female (*n* = 26)									GG vs. GC *P*-value#	GG vs. CC *P*-value#	GC vs. CC *P*-value#
	GG(Gly16Gly) (*n* = 6)			GC(Gly16Arg) (*n* = 13)			CC(Arg16Arg) (*n* = 7)					
	Mean	SD	Range	Mean	SD	Range	Mean	SD	Range			
BMI (kg/m^2)	20.6	1.6	18.8–23.2	20.2	1.5	17.8–22.8	21.6	3.0	18.0–26.6	n.s.	n.s.	n.s.
Body fat (%)	27.8	3.6	24.1–33.5	27.6	3.6	22.2–33.4	29.3	4.3	21.6–36.0	n.s.	n.s.	n.s.

Data are presented as the means with standard deviation (SD). Means are statistically different at $P < 0.05$. #Differences between subjects with the above indicated genotypes of ADRB2, as evaluated using one-way analysis of variance followed by Holm's test. BMI, body mass index.

Table S2. Multiple linear regression analysis using age and BMI as dependent variables in male and female subjects.

	Age (Male, *n* = 26)				BMI (Male, *n* = 26)			
**Independent variables**	**B**	**SE**	**Beta**	***P*-value**	**B**	**SE**	**Beta**	***P*-value**
Sweet taste food preference	−2.367	1.609	−0.307	n.s.	−2.070	5.816	−0.254	n.s.
Salty taste food preference	−2.577	1.661	−0.451	n.s.	2.597	1.680	0.429	n.s.
Sour taste food preference	0.019	1.326	0.004	n.s.	−0.622	1.735	−0.136	n.s.
Bitter taste food preference	1.254	1.148	0.269	n.s.	−2.265	1.384	−0.458	n.s.
High-fat food preference	−0.805	1.000	−0.159	n.s.	2.008	1.044	0.376	n.s.

	Age (Female, *n* = 26)				BMI (Female, *n* = 26)			
**Independent variables**	**B**	**SE**	**Beta**	***P*-value**	**B**	**SE**	**Beta**	***P*-value**
Sweet taste food preference	−0.097	1.011	−0.023	n.s.	0.697	0.661	0.235	n.s.
Salty taste food preference	−1.021	1.058	−0.260	n.s.	−0.986	0.692	−0.354	n.s.
Sour taste food preference	0.746	1.321	0.197	n.s.	−0.273	0.863	−0.102	n.s.
Bitter taste food preference	−0.245	1.170	−0.063	n.s.	−0.002	0.765	−0.001	n.s.
High-fat food preference	−1.101	0.913	−0.282	n.s.	−1.141	0.597	−0.413	n.s.

Using data acquired from the subjects included in the present study, multiple linear analysis was performed to evaluate the relations of age and BMI to each type of food preference. BMI, body mass index; B, partial regression coefficient; SE, standard error; Beta, standard partial regression coefficient; n.s., not significant.
